# Isolation, characterization, and molecular identification of soil bacteria showing antibacterial activity against human pathogenic bacteria

**DOI:** 10.1186/s43141-021-00219-x

**Published:** 2021-08-18

**Authors:** R. Prashanthi, Shreevatsa G.K., Krupalini S., Manoj L.

**Affiliations:** Department of Biotechnology and Genetics, M. S. Ramaiah College of Arts, Science and Commerce, Karnataka 560054 Bengaluru, India

**Keywords:** Soil bacterial communities, 16S rRNA sequencing, Antagonistic activity

## Abstract

**Background:**

The present study dealt with the screening of soil bacteria with antibacterial activity from different locations in Bangalore, India. Antibiotics play the role of self-defense mechanism for the bacteria and are produced as secondary metabolites to protect themselves from other competitive microorganisms. The need for new antibiotics arose as the pathogenic bacteria acquire resistance to various antibiotics meant for treating human diseases. Given the importance of antibiotics of bacterial origin, standard techniques have been used to isolate and characterize the soil bacteria which showed antibacterial activity.

**Results:**

The isolated bacteria were tested against human pathogenic bacteria like *Staphylococcus aureus*, *Escherichia coli*, *Pseudomonas aeruginosa*, and *Klebsiella pneumoniae* by primary and secondary screening methods. The isolates PR1, PR2, and PR3 were confirmed to have antibacterial activity against *S. aureus*, *E. coli*, *P. aeruginosa*, and *K. pneumoniae* by both methods. Studies on the effect of filter sterilization, autoclaving, and proteinase K treatment on culture filtrates showed filter sterilization as the best method. The effect of different carbon and nitrogen sources on the antibacterial activity showed that preference by each isolate differed for carbon and nitrogen requirements. The isolates PR1, PR2, and PR3 were identified as *Bacillus aryabhattai* strain PR-D07, *Arthrobacter humicola* strain PR-F07, and *Neomicrococcus lactis* strain PR-F11 through 16S rRNA sequencing.

**Conclusion:**

Findings from this research work are encouraging and could proceed further to applied aspects. Only 3 bacterial isolates out of 263 isolates from soil samples displayed antibacterial activity against human pathogens *S. aureus*, *E. coli*, *P. aeruginosa*, and *K. pneumoniae*. They were identified as *B. aryabhattai*, *A. humicola*, and *N. lactis* by 16S rRNA studies and all of them are Gram-positive. Each isolate preferred different carbon and nitrogen sources for their enhanced antibacterial activity. Efficacy of the culture filtrates of these isolates was tested by filter sterilization, autoclaving, and proteinase K treatment. Filter-sterilized culture filtrates showed higher antibacterial activity than other treatments. A comparison of the antibacterial activity of culture filtrates and antibiotic streptomycin produced an inhibition zone of 18.5 mm and 15.5 mm respectively. This is the first report on the antibacterial activity of all the 3 bacterial strains (*B. aryabhattai* strain PR-D07, *A. humicola* strain PR-F07, and *N. lactis* strain PR-F11), against all the human pathogens, mentioned earlier. It is also found that the antibiotic factor is proteinaceous as proteinase K considerably reduced the antibacterial activity of the culture filtrates. With the above significant results, these 3 bacteria are considered to be promising candidates for the isolation of new antibacterial agents.

## Background

The existence of a large microbial community in the soil is supported by an enormous group of organic matters in the earth. Most of these microorganisms are bioactive and survive at the top few inches of the agricultural soils [1]. The microorganisms can live in several habitats along with humans and also in the utmost condition such as inside the rocks of the oceanic crust [2], cold temperature [3], hot springs [4], and miles deep in the ocean [5]. Abiotic and biotic factors are involved in regulating the activity and diversification of soil microorganisms. The presence of microbes in soil is based on the existence of ambient conditions provided by the types of vegetation, the texture and chemical nature of the soil, nutrients availability, pH, moisture content, climate, and temperature. The physiology of the soil is also determined by all these conditions as it varies across the same place between different seasons. Further, the dumping of the organic wastes from agricultural fields ensures the availability of the high nutrient content in soil for the growth of the microorganisms [6]. Some bacterial communities in the soil such as *Thiobacillus*, *Rhizobium*, *Nitrosomonas*, *Clostridium*, *Nitrobacter*, *Caulobacter*, *Pseudomonas*, and *Frankia* carry out an essential task in nutrient cycling [7].

During the past decades, an enormous number of bacteria that produce a diverse kind of primary and secondary metabolites, enzymes, antibiotics, and novel compounds, etc., were isolated [8, 9], identified, and exploited by several research groups in human health care, agriculture, and animal husbandry, etc. As these compounds have a unique structure, microorganisms continue to be the essential source of secondary metabolites. The uniqueness of the secondary metabolite is that they act as an antimicrobial agent towards pathogenic bacteria. Antibiotics are not necessary for the growth of bacteria but they help the survival of bacteria [10]. The bacterial community remains the major source of antibiotic production which is widely used in human health care. Each year, nearly 500 antibiotics are discovered, in which most of the antibiotics are obtained from the soil bacteria [11, 12]. Antibiotics are low-molecular-mass (< 1500 kDa) products of secondary metabolism, usually produced during the late growth phase (idiophase) of a relatively small group of microorganisms [10]. Antibiotics play the role of a self-defense mechanism for the bacteria and are produced as secondary metabolites to protect themselves from other competitive microorganisms [13]. Most of the antibiotics which are currently in use are produced from a small group of microorganisms belonging to the genera *Penicillium*, *Streptomyces*, *Cephalosporium*, *Micomonospora*, and *Bacillus* [14]. Members of the species *Bacillus* generally produce polypeptide-type bacteriocins, and these antibiotics are generally effective against several Gram-positive bacteria [15, 16].

India was the largest user of antibiotics with 12·9 × 10^9^ units (10·7 units per person) as per the data available for 2010 [17]. The demand for bacterial antibiotics continues to increase globally owing to the pathogenic bacteria acquiring resistance to existing antibiotics and many antibiotics proved that they are no longer potent against the infections [18, 19]. Multidrug resistance in bacteria raised serious concerns among pharmaceutical and healthcare researchers. It puts greater pressure among researchers to find alternative antibacterial substances that can be used for use in clinics, food preservation, and dairy products [20, 21]. The indiscriminate use of antibiotics and their improper disposal lead to drug resistance in pathogenic bacteria and the antibiotics become less effective for their use. At present, modern medicine is facing a tremendous challenge to combat the antibiotic resistance acquired by several pathogenic species. Antibiotics are aimed to inhibit the growth or kill the microbes that cause infectious diseases and drug resistance is considered a threat to health security [22]. To survive the adverse environment, bacteria evolve mechanisms to modify or acquire new genes through natural ways and eliminate the effectiveness of drugs [23].

An incomplete dose of any prescribed antibiotic also facilitates the pathogenic bacteria to develop resistance. This necessitates a situation to find new antibiotics to meet the drug resistance challenges. Considering these in mind, this study was focused on isolation and characterization of antibiotic-producing bacteria from the soil in 7 different sites like the garden, the playground, near the canteen, and near the sewage sump covered with vegetation. In this study, we have found that some soil bacteria displayed antibacterial activity and they are characterized and identified using molecular methods.

## Method

### Collection of soil sample

The soil collection site is located in and around with a lot of native vegetation at an altitude of 949 m with a latitude and longitude of 12.87° N, 77.59° E. The types of soil in the location consist of red laterite and fine loamy to clayey. The debris from the soil surface was removed before the collection of soil samples. The soil was dug into 5–10-cm depth. About 20 g of the soil samples were collected and stored in an icebox before transporting to the laboratory.

### Pathogenic bacteria and culture conditions

The standard serial dilution technique was used for the isolation of bacteria from soil samples collected from 7 different sites like the garden, the playground, near the canteen, near the sewage sump, near the biotechnology department, near RIT, and RUAPS. One gram of soil sample was mixed with l0 ml of sterile water and serially diluted (10^−1^ to 10^−4^). From the serially diluted soil sample, 100 μl was mixed with warm nutrient agar medium and poured into Petri plates. Natamycin (Sigma-Aldrich, USA) at 20 μg/ml was amended with a molten nutrient agar medium at 50 C to prevent fungal growth [24, 25]. After 48 h, the plates had a lawn of mixed bacterial colonies. The individual colonies were picked using sterile toothpicks and streaked onto fresh nutrient agar plates to get pure cultures. The pure culture was stored and used for testing antibacterial activity against human pathogenic bacteria *Staphylococcus aureus* (MTCC 96), *Escherichia coli* (MTCC 739), *Pseudomonas aeruginosa* (MTCC 741), and *Klebsiella pneumoniae* (MTCC 3040). The concentration of all pathogenic bacteria was adjusted to obtain the OD = 0.8 using a UV/Vis spectrophotometer at 600 nm.

### Primary screening

A total of 263 bacterial colonies were isolated from soil samples collected from 7 different sites. Initial screening of the 263 soil bacteria for antagonistic activity was done in an in vitro condition against pathogenic bacteria like *Staphylococcus aureus* (MTCC 96), *Escherichia coli* (MTCC 739), *Pseudomonas aeruginosa* (MTCC 741), and *Klebsiella pneumoniae* (MTCC 3040) through perpendicular streaking [26] and seed overlay method [27]. The bacteria from the soil sample were individually streaked as a single straight line through the central point of the nutrient agar plates. All the pathogenic bacteria were perpendicularly streaked to the soil bacteria [26]. The plates were incubated for 24 h to find any inhibition zone between soil bacteria and the pathogenic bacteria. The bacterial strains showing an inhibition zone against test pathogens were chosen for secondary screening.

### Seed overlay method

The isolated soil bacteria were inoculated using a sterile toothpick in a nutrient agar plate and incubated for 48 h followed by the addition of 2 ml of chloroform to arrest the growth of the inoculated bacteria. The plates were incubated for 1 h to ensure only the secondary metabolites from the inoculums remain active on the nutrient agar plate. The plates were kept open for 20 min for the evaporation of the chloroform. Then, 100 μl of each pathogenic bacterium (OD = 0.8) was mixed with 2 ml of nutrient broth and mixed thoroughly. The medium was transferred to the above agar plate and incubated for 24 h. The activity of the secondary metabolites against pathogenic bacteria was indicated by the diameter of the inhibition zone [27]. The bacteria which produced the inhibition zone were chosen for secondary screening.

### Secondary screening to confirm antibacterial activities

All the active bacteria selected from the primary screening method were grown separately in Nutrient broth at 30 °C under shaking conditions. After 24 h, the nutrient broth with cells was adjusted to get an OD of 0.8 at 600 nm using a UV/Visible spectrophotometer (SYSTRONICS, India, Model: AU-2702). It was centrifuged at 5000 × g for 10 min in a centrifuge (Remi, Model: CPR-24Plus) and cell free-culture filtrate was collected and stored at 4 °C.

The minimum inhibitory concentrations (MICs) of the culture filtrate were determined by using the Agar Well Diffusion method. Nutrient agar plates were inoculated with 100 μl of the pathogenic organism by the spread plate method. Using a 6-mm-diameter cork borer, 2 wells were made in agar plates at equal distances and the wells were filled with 50 μl, and 100 μl, of cell-free culture filtrates of PR1, PR2, and PR3 separately. Then, the filter paper disc about 6 mm in diameter impregnated with streptomycin (20 μl,) was placed on the agar surface. Streptomycin discs were used as a positive control. The agar plates were incubated for 2 days at 30 °C for bacterial growth. Antibacterial activity of culture filtrate was determined by measuring the zone of inhibition (Kirby-Bauer Test) around the well [28].

### Effect of filter sterilization, autoclaving, and proteinase K treatment on culture filtrate

To test the efficacy of culture filtrates of PR1, PR2, and PR3 as a sustainable antibacterial agent, they were subjected to (a) filtering through a 0.45-μm Millipore filter (b), autoclaving at 121 °C for 20 min, and (c) treating with proteinase K (0.02 mg/ml) at 50 °C for 1 h. Antibacterial activity was tested using the following: (1) crude cell-free culture filtrate, (2), filter-sterilized culture filtrate, (3), filter-sterilized + heat-sterilized, and (4), filter-sterilized + proteinase K. Inhibition zone for each treatment was measured and presented.

### Effect of carbon and nitrogen sources on antibacterial activity

The effect of different carbon and nitrogen sources on the antibacterial activity of the culture filtrates of PR1, PR2, and PR3 was studied. Fifty milliliters of the synthetic medium amended with various carbon (1%) and nitrogen (0.3%) sources was distributed into each 250-ml Erlenmeyer flask and sterilized. The composition of the synthetic medium was sucrose 10 g, K_2_HPO_4_ 1.2 g, KH_2_PO_4_ 0.8 g, MgSO4 7H_2_O 0.2 g, NH_4_NO_3_ 0.3 g, water 1000 ml, and pH 6.8–7.00. Arabinose, fructose, galactose, glucose, lactose, maltose, mannitol, and sucrose were used as carbon sources. Casein, NH_4_Cl, NH_4_NO_3_, NaNO_3_, NH_4_H_2_PO_4_, KNO_3_, (NH4)_2_SO4, and urea were used as nitrogen sources. After inoculation with PR1, PR2, and PR3, the flasks were incubated at 30 °C for 48 h. under shaking conditions. At the end of 48 h, the liquid cultures of PR1, PR2, and PR3 were centrifuged at 5000 × g in a centrifuge. Cell-free culture filtrates were collected and stored at 4 °C.

### Morphological and biochemical analysis of bacteria

Morphological and biochemical characterizations of the bacteria that showed antibacterial activity was carried out using standard techniques described in Bergey’s Manual of Determinative Bacteriology. Bacteria grown for 24 h in the nutrient broth were used for Gram staining and biochemical characterization.

### Molecular identification of bacteria

Overnight-grown cultures of PR1, PR2, and PR3 were used for DNA isolation. About 2.0 ml of bacterial suspension was transferred to a microcentrifuge tube and centrifuged for 2 min at 10,000 × g to collect the pellet. The above process was repeated twice with 2.0 ml of bacterial suspension to obtain a sufficient number of cells. The cells were washed with 0.9% saline and 0.2 ml protease was added to digest and remove the protein and cellular materials to release the genomic DNA. The centrifuged tubes were inverted 5–6 times and kept in a boiling water bath for 1 h at 55 °C. After 1 h, 0.1 ml of the DNA Salt solution was added and centrifuged for 5 min at 5,000 × g. Finally, 0.8 ml of the precipitated solution was added slowly and the centrifuge tube inverted several times to mix the components. 70% ethanol was used to wash the cells. The collected DNA were dried and suspended in TE buffer and stored at 4 °C. The quality and integrity of the isolated genomic DNA were quantified at wavelength 260 and 280 nm using a spectrophotometer. The purity of the extracted DNA was checked on 08% agarose gel.

For the amplification of the 16S rRNA gene fragments the universal primers were used (forward primer 5′-AGAGTTTGATCCTGGCTCAG-3′ and reverse primer 5′-GGTTACCTTGTTACGACT-3′). PCR parameters were initial denaturation at 95 °C for 5 min; followed by 30 cycles of denaturation at 94 °C for 1 min; annealing at 54 °C for 2 min; extension at 72 °C for 2 min; final extension at 72 °C for 5 min. The amplified PCR products were electrophoresed on an agarose gel [29].

Amplified 16S rRNA gene fragments were purified and sequenced using a DNA sequencing service. The obtained 16S rRNA gene sequences were uploaded to the Basic Local Alignment Search Tool (BLAST) to identify nucleotide sequence matching with the reference sequences.

## Results

### Soil sample

The soil samples from 7 sites were serially diluted and spread on Nutrient agar plates and incubated at 30 C for 3 days. A total of 263 bacterial colonies were isolated from all the sites mentioned earlier. Individual colonies were picked and inoculated into Petri plates containing nutrient agar medium and incubated at 30 °C. A maximum number of colonies were noticed in soil samples collected from the garden, near the sewage sump, and near the canteen compared to other soil samples (Table 1).

**Table 1:** Bacteria from soil samples

Soil sample (location)	Dilution	CFU /ml	No. of colonies	Antibacterial activity
Garden	10^−3^	9.8 × 10^6^	69	PR1, PR2
Playground	10^−2^	4.5 × 10^6^	15	Nil
Near canteen	10^−3^	8.7 × 10^5^	48	Nil
Near sewage sump	10^−3^	9.2 × 10^6^	54	PR3
Near biotech dept.	10^−3^	4.5 × 10^6^	45	Nil
Near RIT	10^−3^	3.5 × 10^5^	12	Nil
Near RUAPS	10^−3^	5.5 × 10^6^	20	Nil
Total			263	3

### Primary screening for antibacterial activity

All the 263 bacterial isolates were tested for their antibacterial activity against pathogenic bacteria *S. aureus*, *E. coli*, *P. aeruginosa*, *and K. pneumoniae* using primary screening. The results of the primary screening showed that only 2 isolates (PR1 and PR2) from garden soil and one isolate (PR3) from the soil collected near the sewage sump had antibacterial activity against all the tested bacteria (Table 2). The isolates PR1, PR2, and PR3 were selected for secondary screening.

**Table 2:** Antibacterial activity against pathogenic bacteria by primary screening

Isolates	*S. aureus*	*E. coli*	*P. aeruginosa*	*K. pneumoniae*
PR1	**+**	**+**	**+**	**+**
PR2	**+**	**+**	**+**	**+**
PR3	**+**	**+**	**+**	**+**

### Secondary screening for antibacterial activity 

The 3 isolates PR1, PR2 & PR3 which were confirmed to have antibacterial activity through primary screening were subjected to secondary screening for further confirmation of their activity against human pathogens *S. aureus*, *E. coli*, *P. aeruginosa*, and *K. pneumoniae*. All culture filtrates at 100 μl showed a maximum inhibition zone except *E. coli* indicating that Gram (+) bacteria are susceptible to antibiotics than Gram (−) bacteria. The filter-sterilized culture filtrate of PR1 showed a maximum zone of inhibition with 15.5 mm, 10.0 mm, 15.0 mm, and 14.0 mm compared to PR2 and PR3 against the tested pathogens (Table 3). Although all the isolates showed antibacterial activity against all pathogenic bacteria, autoclaved and proteinase K-treated culture filtrates lost their activity by 30 to 40% (Table 4) indicating that filter sterilization of crude culture filtrate is the best option for testing antibacterial activity. The inhibition zone for standard antibiotic streptomycin was 17.5 to 18.5 mm depending on the pathogens.

**Table 3:**
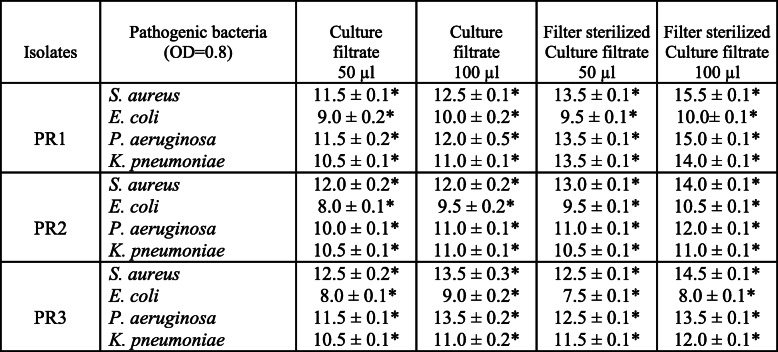
Antibacterial activity of culture filtrate of PR1, PR2, and PR3 against pathogenic bacteria

Results are expressed as antagonistic activity (mm) of the isolate bacteria against pathogenic compared to control (mean ± SD, *n* = 3). Values significantly different from control if *ρ < 0.05 as analyzed by Student’s *t*-test. Control value for all the pathogenic bacteria 6 mm

PR1*—Bacillus aryabhattai* strain PR-D07, PR2*—Arthrobacter humicola* strain PR-F07, PR*—Neomicrococcus lactis* strain PR-F11

**Table 4:**
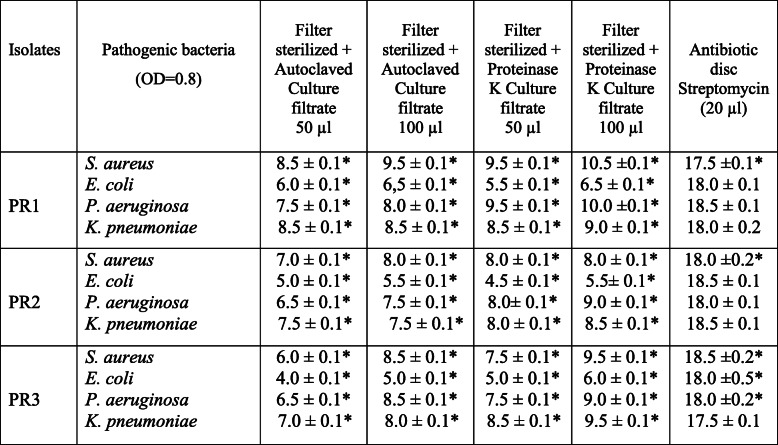
Antibacterial activity of culture filtrate of PR1, PR2, and PR3 against pathogenic bacteria

Results are expressed as antagonistic activity (mm) of the isolate bacteria against pathogenic compared to control (mean ± SD, *n* = 3). Values significantly different from control if *ρ < 0.05 as analyzed by student t-test. Control value for all the pathogenic bacteria 6 mm

PR1*—Bacillus aryabhattai* strain PR-D07, PR2*—Arthrobacter humicola* strain PR-F07, PR3*—Neomicrococcus lactis* strain PR-F11

### Effect of carbon and nitrogen sources on antibacterial activity

Studies on the effect of different carbon and nitrogen source on the antibiotic activity revealed that Glycerol and Urea were the preferred carbon and nitrogen source for the isolate PR1. Isolate PR2 preferred Glucose and Casein while PR3 preferred sucrose and casein. Nutrient preference varied among all the isolates tested. Among the 4 test organisms, the inhibition zone for the Gram-negative bacterium *E. coli* was smaller than all other bacteria (Table 5). Commercial antibiotic streptomycin showed a larger inhibition zone than all the culture filtrates. A synthetic medium with sucrose and ammonium nitrate as carbon and nitrogen source was used as a control.

**Table 5:** Effect of carbon and nitrogen sources on antibacterial activity by PR1, PR2, and PR3

Pathogens (OD = 0.8)	Carbon source	Inhibition zone (mm dia) PR1	Inhibition zone (mm dia) PR2	Inhibition zone (mm dia) PR3	Nitrogen source	Inhibition zone (mm dia) PR1	Inhibition zone (mm dia) PR2	Inhibition zone (mm dia) PR3
*S. aureus*	Glucose	**++++**	**++**	**++**	Casein	**++**	**+++**	**++**
*E. coli*		**++**	**++**	**++**		**++**	**++**	**++**
*P. aeruginosa*		**++++**	**++**	**++**		**++**	**+++**	**++**
*K. pneumoniae*		**++++**	**++**	**++**		**++**	**+++**	**++**
*S. aureus*	Sucrose	**++**	**++**	**+++**	NH_4_Cl	**++**	**++**	**++**
*E. coli*		**++**	**++**	**++**		**++**	**++**	**++**
*P. aeruginosa*		**++**	**++**	**+++**		**++**	**++**	**++**
*K. pneumoniae*		**++**	**++**	**+++**		**++**	**++**	**++**
*S. aureus*	Glycerol	**++**	+++	**++**	NH_4_NO_3_	**++**	**++**	**++**
*E. coli*		**++**	**++**	**++**		**++**	**++**	**++**
*P. aeruginosa*		**++**	**+++**	**++**		**++**	**++**	**++**
*K. pneumoniae*		**++**	**+++**	**++**		**++**	**++**	**++**
*S. aureus*	Fructose	**++**	**++**	**++**	NaNO_3_	**++**	**++**	**++**
*E. coli*		**++**	**++**	**++**		**++**	**++**	**++**
*P. aeruginosa*		**++**	**++**	**++**		**++**	**++**	**++**
*K. pneumoniae*		**++**	**++**	**++**		**++**	**++**	**++**
*S. aureus*	Galactose	**++**	**++**	**++**	Urea	**++++**	**++**	**++**
*E. coli*		**++**	**++**	**++**		**++**	**++**	**++**
*P. aeruginosa*		**++**	**++**	**++**		**++++**	**++**	**++**
*K. pneumoniae*		**++**	**++**	**++**		**++++**	**++**	**++**
*S. aureus*	Lactose	**++**	**++**	**++**	NH_4_H_2_PO_4_	**++**	**++**	**+++**
*E. coli*		**++**	**++**	**++**		**++**	**++**	++
*P. aeruginosa*		**++**	**++**	**++**		**++**	**++**	**+++**
*K. pneumoniae*		**++**	**++**	**++**		**++**	**++**	**+++**
*S. aureus*	Maltose	**++**	**++**	**++**	KNO_3_	**++**	**++**	**++**
*E. coli*		**++**	**++**	**++**		**++**	**++**	**++**
*P. aeruginosa*		**++**	**++**	**++**		**++**	**++**	**++**
*K. pneumoniae*		**++**	**++**	**++**		**++**	**++**	**++**
*S. aureus*	Arabinose	**++**	**++**	**++**	NH4SO_4_	**++**	**++**	**++**
*E. coli*		**++**	**++**	**++**		**++**	**++**	**++**
*P. aeruginosa*		**++**	**++**	**++**		**++**	**++**	**++**
*K. pneumoniae*		**++**	**++**	**++**		**++**	**++**	**++**
*S. aureus*	Control sucrose	**++**	**++**	**++**	Control NH_4_NO_3_	**++**	**++**	**++**
*E. coli*		**++**	**++**	**++**		**++**	**++**	**++**
*P. aeruginosa*		**++**	**++**	**++**		**++**	**++**	**++**
*K. pneumoniae*		**++**	**++**	**++**		**++**	**++**	**++**

**++** 5–10 mm, +++ 10–15 mm, ++++ above 15 mm

PR1*—Bacillus aryabhattai* strain PR-D07, PR2*—Arthrobacter humicola* strain PR-F07, PR3*—Neomicrococcus lactis* strain PR-F11

### Morphological and biochemical characterization of antagonistic bacteria

The morphological and biochemical characterization of bacterial isolates PR1, PR2, and PR3 which showed antibacterial activity were done, and results are tabulated (Table 6).

**Table 6:** The morphological and biochemical characterization of bacterial isolates

Test	PR1	PR2	PR3
Gram Stain	+	+	+
Motility	+	+	−
Colony color	Opaque and off-white	Cream	Yellow pigmented colonies
Cell shape	Rod-shaped	Rod-shaped	Cocci
Spore forming	+	−	−
Oxidase	+	−	−
Catalase	+	+	+
Amylase	+	−	−
Gelatinase	−		−
Phosphatase	−	+	+
Tryptophane deaminase	−	−	−
Arginine dihydrolase	−	−	−
Lysine decarboxylase	−	−	−
Ornithine decarboxylase	−	−	−
Glutamate decarboxylase	−	−	−
Voges-Proskauer	+	−	−
Nitrate reduction	−	−	+
Hydrolysis of Casein	+	−	+
Hydrolysis of starch	−	−	−
Hydrolysis of urea	−	−	+
Indole Production	−	−	−

### Molecular identification of bacteria

The isolation of genomic DNA was carried out to obtain pure DNA from the isolates PR1, PR2, and PR3. The 16sRNA was amplified from the isolated DNA sample using PCR. 1.2% of agarose gel was used to verify the amplified products which showed a fragment of 1.5 kb*.*

The amplified 16s r RNA of PR1, PR2, and PR3 were subjected to purification and sequencing. The sequences of PR1, PR2, and PR3 were submitted to NCBI. The accession numbers of the sequence PR1, PR2, and PR3 are MT453908—*Bacillus aryabhattai* strain PR-D07, MT453911—*Arthrobacter humicola* strain PR-F07, and MT453912—*Neomicrococcus lactis* strain PR-F11.

The sequence of MT453908—*Bacillus aryabhattai* strain PR-D07

TATCCCCGGGAGCCCGACCCGGCGCCGCAAGTCGGAACCAGGTACCCGTATAGTTTGATCCTGGCTCAGG

ATGAACGCTGGCGGCGTGCCTAATACATGCAAGTCGAGCGAACTGATTAGAAGCTTGCTTCTATGACGTT

AGCGGCGGACGGGTGAGTAACACGTGGGCAACCTGCCTGTAAGACTGGGATAACTTCGGGAAACCGAAGC

TAATACCGGATAGGATCTTCTCCTTCATGGGAGATGATTGAAAGATGGTTTCGGCTATCACTTACAGATG

GGCCCGCGGTGCATTAGCTAGTTGGTGAGGTAACGGCTCACCAAGGCAACGATGCATAGCCGACCTGAGA

GGGTGATCGGCCACACTGGGACTGAGACACGGCCCAGACTCCTACGGGAGGCAGCAGTAGGGAATCTTCC

GCAATGGACGAAAGTCTGACGGAGCAACGCCGCGTGAGTGATGAAGGCTTTCGGGTCGTAAAACTCTGTT

GTTAGGGAAGAACAAGTACAAGAGTAACTGCTTGTACCTTGACGGTACCTAACCAGAAAGCCACGGCTAA

CTACGTGCCAGCAGCCGCGGTAATACGTAGGTGGCAAGCGTTATCCGGAATTATTGGGCGTAAAGCGCGC

GCAGGCGGTTTCTTAAGTCTGATGTGAAAGCCCACGGCTCAACCGTGGAGGGTCATTGGAAACTGGGGAA

CTTGAGTGCAGAAGAGAAAAGCGGAATTCCACGTGTAGCGGTGAAATGCGTAGAGATGTGGAGGAACACC

AGTGGCGAAGGCGGCTTTTTGGTCTGTAACTGACGCTGAGGCGCGAAAGCGTGGGGAGCAAACAGGATTA

GATACCCTGGTAGTCCACGCCGTAAACGATGAGTGCTAAGTGTTAGAGGGTTTCCGCCCTTTAGTGCTGC

AGCTAACGC

The sequence of MT453911—*Arthrobacter humicola* strain PR-F07

TCAAACTCCCTTAGATTTGATCCTGGCTCAGGACGAACGCTGGCGGCGTGCTTAACACATGCAAGTCGAA

CGATGATCCGGTGCTTGCACCGGGGATTAGTGGCGAACGGGTGAGTAACACGTGAGTAACCTGCCCTTGA

CTCTGGGATAAGCCTGGGAAACCGGGTCTAATACCGGATATGACTTCCTGCCGCATGGTGGGGGGTGGAA

AGATTTTTTGGTTTTGGATGGACTCGCGGCCTATCAGCTTGTTGGTGGGGTAATGGCCTACCAAGGCGAC

GACGGGTAGCCGGCCTGAGAGGGTGACCGGCCACACTGGGACTGAGACACGGCCCAGACTCCTACGGGAG

GCAGCAGTGGGGAATATTGCACAATGGGCGCAAGCCTGATGCAGCGACGCCGCGTGAGGGATGACGGCCT

TCGGGTTGTAAACCTCTTTCAGCAGGGAAGAAGCGGAAGTGACGGTACCTGCAGAAGAAGCGCCGGCTAA

CTACGTGCCAGCAGCCGCGGTAATACGTAGGGCGCAAGCGTTGTCCGGAATTATTGGGCGTAAAGAGCTC

GTAGGCGGTTTGTCGCGTCTGCTGTGAAAGCCCGGGGCTCAACCCCGGGTCTGCAGTGGGTACGGGCAGA

CTGGAGTGCAGTAGGGGAGACTGGAATTCCTGGTGTAGCGGTGAAATGCGCAGATATCAGGAGGAACACC

GATGGCGAAGGCAGGTCTCTGGGCTGTAACTGACGCTGAGGAGCGAAAGCATGGGGAGCGAACAGGATTA

GATACCCTGGTAGTCCATGCCGTAAACGTTGGGCACTAGGTGTGGGGGACATTCCACGTTTTCCGCGCCC

GTAGCTAACGCCC

The sequence of MT453912—*Neomicrococcus lactis* strain PR-F11

GAGAATTCCACGTTTTTCCGCGCCGTAGCTAACGCATTAAGTGCCCCGCCTGGGGAGTACGGCCGCAAGG

CTAAAACTCAAAGGAATTGACGGGGGCCCGCACAAGCGGCGGAGCATGCGGATTAATTCGATGCAACGCG

AAGAACCTTACCAAGGCTTGACATGGGCCGGATCGCCGCAGAAATGCGGTTTCCCTTCGGGGCCGGTTCA

CAGGTGGTGCATGGTTGTCGTCAGCTCGTGTCGTGAGATGTTGGGTTAAGTCCCGCAACGAGCGCAACCC

TCGTTCTATGTTGCCAGCGGTTCGGCCGGGGACTCATAGGAGACTGCCGGGGTCAACTCGGAGGAAGGTG

GGGACGACGTCAAATCATCATGCCCCTTATGTCTTGGGCTTCACGCATGCTACAATGGCCGGTACAAAGG

GTTGCGATACTGTGAGGTGGAGCTAATCCCAAAAAGCCGGTCTCAGTTCGGATTGAGGTCTGCAACTCGA

CCTCATGAAGTCGGAGTCGCTAGTAATCGCAGATCAGCAACGCTGCGGTGAATACGTTCCCGGGCCTTGT

ACACACCGCCCGTCAAGTCACGAAAGTTGGTAACACCCGAAGCCGGTGGCCTAACCCTTTTGGGAGGGAG

CCGTCGAAGGTGGGACCGGCGATTGGGACTAAGTCGTAACAAGGTAACCGATAAGG

### Phylogeny of PR1, PR2, and PR3

**Fig. 1:**
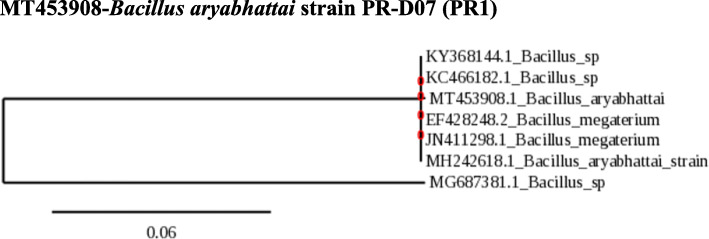
Neighbor-joining tree showing the phylogenetic relationships of 16S rRNA gene sequences of *B. aryabhattai*. The scale bar indicates evolutionary distance

**Fig. 2:**
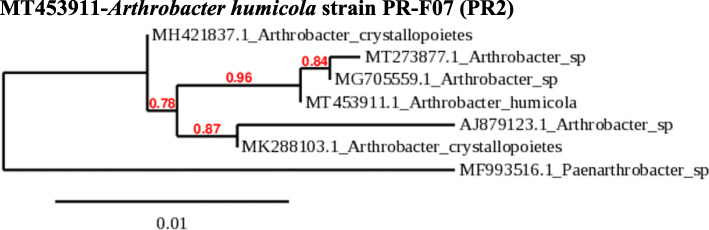
Neighbor-joining tree showing the phylogenetic relationships of 16S rRNA gene sequences of *A. humicola*. The scale bar indicates evolutionary distance

**Fig. 3:**
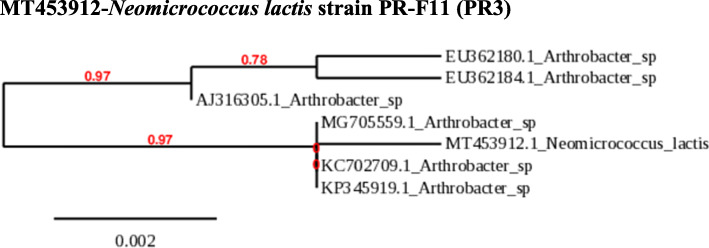
Neighbor-joining tree showing the phylogenetic relationships of 16S rRNA gene sequences of *N. lactis*. The scale bar indicates evolutionary distance

The obtained sequences were compared with sequences in the Genbank nucleotide database. The identification of the species is done with the phylogenetic analysis neighbor with 98 – 100 % similarity. Phylogenetic analysis and sequence alignment were carried out using the http://www.phylogeny.fr/simple_phylogeny.cgi. This indicates that collected strains are *B. aryabhattai* (PR1*)*, *A. humicola* (PR2), and *N. lactis* (PR3) (Figs. 1, 2, and 3).

## Discussion

Soil is found to be a rich source for various types of bacterial communities when compared to other environments and they are very well adapted to constantly varying soil environments. Competition among them for survival necessitates producing antibacterial compounds to eliminate the competitor. This is one of the reasons why soil bacteria are preferred for screening of antibacterial activity [30]. Numerous scientists have selected soil for the isolation of countless antibiotic-producing bacteria. Arifuzzaman et al. [31], a revealed 20 soil bacterial strains were active against the pathogenic microbes. Denizci [32] isolated 356 *Streptomyces* isolates from the soils in several regions of Turkey and screened for antibacterial activity. Dehnad et al. [33] have isolated 150 actinomycetes from soil samples of West of Iran for screening antibacterial activity against the test pathogens. Falkinham et al. [34] reported that soil bacteria form the basis for the production of nearly 500 antibiotics each year. The demand for bacterial antibiotics continues to increase globally because the pathogenic bacteria continue to acquire resistance to the antibiotics and many antibiotics proved that they are no longer potent against the infections [18, 19]. It is reported that some *Staphylococcus* spp., *Streptococcus* spp., pseudomonads, and Enterobacteria, responsible for several human health issues, have developed resistance to many antibiotics [35].

This study is aimed to isolate soil bacteria that exhibit antibacterial activity. Out of 263 bacterial colonies isolated from soil samples, only 3 had antibacterial potential. The 3 active bacterial strains (*B. aryabhattai* strain PR-D07, *A. humicola* strain PR-F07, and *N. lactis* strain PR-F11), isolated and identified through molecular methods in this study are the first report on their antibacterial activity against all the human pathogens mentioned earlier. Any reports of antibacterial activity by these isolates and their preferential carbon and nitrogen source are not available so far.

Nike et al. [36] and Kaur et al. [37] have used the primary and secondary screening methods as done in our work for the isolation and screening of bacterial isolates. Many scientists have adopted the agar well diffusion method for secondary screening of bacteria using cell-free culture filtrates [38–40]. Similar studies on different bacteria were carried out by Rafiq et al. [41] and their study suggested that most of the species belonging to the genus *Bacillus* are potential for the production of antibiotics. Our study also found that one of the active bacteria was *B. aryabhattai*.

In this study, all culture filtrates at 100 μl inhibited the growth of all bacterial pathogens listed earlier. It shows that PR1, PR2, and PR3 displayed broad-spectrum activity against both the Gram (+) and Gram (−) bacteria. However, the zone of inhibition for *S. aureus*, *P. aeruginosa*, and *K*, *pneumoniae* was bigger than the inhibition zone for *E. coli* indicating that Gram (+) bacteria are more vulnerable to antibiotics than Gram (−) bacteria. The difference in membrane constituents of Gram (+) and Gram (−) is responsible for the difference in susceptibility, outer polysaccharide membrane possessed by Gram (−) did not permit the entry of lipophilic solutes whereas the peptidoglycan layer of Gram (+) bacteria is not an effective barrier [42]. In their work, Ray et al. [43] reported that culture filtrates of *B. aryabhattai* LS11, isolated from wetland soils failed to inhibit *E.coli* at all concentrations.

In our studies, the filter-sterilized culture filtrate of *B. aryabhattai* (PR1) showed maximum antibacterial activity against all tested pathogens which is similar to the results of Yoshida et al. (38), who used the filter-sterilized culture filtrate of *B. amyloliquefaciens* against many bacteria. However, Onajobi et al. [44] reported that the culture filtrate of *B. aryabhattai* KNUC205 isolated from farmland soil showed antibacterial activity only against *P. aeruginosa* and failed to show any activity against *S. aureus*, *E. coli*, and *K. pneumoniae*, tested by them.

Autoclaving and proteinase K treatment of culture filtrate reduced the antibacterial activity against all tested pathogens. Reduction in the antibacterial activity of culture filtrate as a result of proteinase K treatment indicated that the antibacterial principle is proteinaceous [45]. Members of the genus *Bacillus* were found to produce different types of peptides which are responsible for the broad spectrum of antibacterial activity against pathogenic bacteria [46]. Proteins, whether they are a simple or complex group of polypeptides make pathways with several enzymatic steps using polyketide synthases and peptide synthetases to produce antibiotics (10). Meng et al. [47] and Siahmashteh et al. [48] have shown that *Bacillus* species are found to be a robust source of antibiotics against various pathogens.

The antibacterial activity shown by soil bacteria is governed by the nutritional status of the soil which includes carbon and nitrogen sources. Therefore, optimization of essential substrates is required for the production of a high level of antibacterial compounds [14]. The effect of different carbon and nitrogen sources on antibacterial activity was studied as these have significant effects on bacterial metabolism. The priority of nutritional substrates widely varies among every isolate. In our studies, 3 different isolates preferred 3 different carbon and nitrogen sources. *B. aryabhattai* strain PR-D07 (PR1), *A. humicola* strain PR-F07 (PR2), and *N. lactis* strain PR-F11 (PR3) preferred glucose, glycerol, and sucrose as carbon sources respectively. Hence, there is a difficulty in arriving at a common formula regarding the nutritional requirement for all isolates. Our results revealed that 1% glucose as the sole carbon source stimulated increased antibacterial activity more than other sugars in the case of the isolate *B. aryabhattai* strain PR-D07 (PR1). These results were in agreement with the earlier reports which stated that 1% glucose was the optimum carbon source for antibiotic production in *Streptomyces* sp. [49, 50] in *Brevibacillus laterosporus* EA62 [51] and *Bacillus subtilis* [52]. However, Pandey et al. [53] found that 2% of Dextrose is the preferred source by *S. kanamyceticus.* Dunia et al. [54] reported enhanced production of antibiotics by wheat bran as a carbon source in *Streptomyces* sp. whereas Alev Usta et al. [51] found that the antibiotic activity of *Brevibacillus laterosporus* EA62 was repressed by wheat bran but a higher growth rate was observed. Zhang et al. [55] found enhanced antibacterial activity if sucrose was used for the growth of *B. amyloliquifaciens*. El-Banna [56] had tested 5 different strains of *B. megaterium*, and their preference to carbon source significantly varied for enhanced antibacterial activity. According to their results, *B. megaterium* NB-3 and NB-6 utilized Glycerol, *B. megaterium* NB-4 and NB-5 used Glucose, and *B. megaterium* NB-7 preferred Fructose for enhanced antibiotic activity against tested bacteria. Glycerol as a carbon source displayed higher antibacterial activity by *B. firmus* and *B. circulans*, starch for *B. stearothermophilus*, and an unknown strain [57].

Similarly, preference for nitrogen source also differed among all the 3 isolates tested in the present study. *B. aryabhattai* strain PR-D07 (PR1) showed maximum antibacterial activity using urea than all other nitrogen sources. *A. humicola* strain PR-F07 (PR2) preferred casein and *N. lactis* strain PR-F11 (PR3) used NH_4_H_2_PO_4_ for antibacterial activity. Although the isolate *B. aryabhattai* strain PR-D07 (PR1) preferred urea, Zhang et al. [55] reported urea led to the loss of antibacterial activity in *B. amyloliquifaciens* and it preferred NH_4_Cl as the best nitrogen source for antibiotic activity.

In a study by Dunia et al. [54], yeast extract, ammonium sulfate, and beef extract as a supplement to wheat bran produced 249 U/g, 240 U/g, 220 U/g of antibiotic respectively by *Streptomyces* sp. AS4 which is comparatively higher than the wheat bran alone. Oskay [58] and Al-Ghazali and Omron, [59] have reported peptone as the excellent nitrogen source for *Streptomyces* sp. and *Streptomyces* sp. KGG32 respectively for antibiotic production.

Morphological and biochemical characterization of isolates PR1, PR2, and PR3 were carried out as it is a tool for preliminary identification of bacteria, and it is a conventional method followed by microbiologists all over the world [37, 60]. For the past several decades, most laboratories adopt microscopic identification and biochemical characterization to identify the bacteria [61]. As the bacteria do not have sufficient morphological features to confirm their identity, several procedures have been formulated based on their nutrition, metabolic activities, metabolic products, or enzymatic reactions which help in grouping and identifying the bacteria up to genus and species level [62, 63]. The Gram staining results showed that the isolates PR1, PR2, and PR3 are Gram (+). The biochemical tests carried out in our studies matched with these isolates.

Phylogenetic analysis and sequence alignment of the isolates PR1, PR2, and PR3 revealed them as *B. aryabhattai*, *A. humicola*, and *N.lactis*. The isolate *B. aryabhattai* PR-D07 (PR1) displayed a high antibacterial activity than other isolates. Several reports confirmed the fact that many species of the genus *Bacillus* are potential antibiotic producers [41]. Ours is the first report on the antibacterial activity of *B. aryabhattai* against human pathogens *S. aureus*, *E. coli*, *P. aeruginosa*, and *K. pneumoniae*.

Apart from its antibacterial property, *B. aryabhattai* was reported to be the producer of many value-added products. Yaraguppi et al. [64] have reported that *B. aryabhattai* could be a promising candidate for exploring the production of biosurfactants relevant to the pharmaceutical industry. Paz et al. [65] have obtained several value-added products from *B. aryabhattai* by using different media. They suggested this bacterium could be used to degrade lignocellulose wastes and treating dyes from the textile industry. It indicates that *B. arybhattai* could be a potential organism to study in detail. The antibacterial activity shown by *B. aryabhattai* (isolate PR1) would be as effective as that of the commercial antibiotic provided further purification and characterization of antibiotic factor is carried out.

In the present work, the isolate PR2 was identified as *A. humicola* PR-F07 exhibited antibacterial activity on all test bacteria; however, its activity was comparatively lower than *B. aryabhattai* PR-D07vNo report is available on the antibiotic activity of *A. humicola* and ours is the first report of antibiotic activity by *A. humicola*. However, Munaganti et al. [66] have reported that modified yeast extract malt extract dextrose broth enhanced the bioactive compound formation in *A. kerguelensis*. According to their experimental results, lactose and peptone were the best carbon and nitrogen sources. Bacteria of the genus *Arthrobacter* are commonly found in the soil environment and Kageyama et al. [67] isolated this bacteria from the paddy field. The bioflocculants produced by *A. humicola* were reported to be used for sewage wastewater treatment replacing the chemically produced flocculants [68].

The isolate PR3 which showed antibacterial activity was identified as *N. lactis* PR-F11. This new species was proposed and described by Prakash et al. [69]. There is no report on the production of antibiotics by *N. lactis* so far. Ours is the first report on antibacterial activity by *N. lactis.* Biscupiak et al. [70] reported that *Micrococcus luteus* produced an antibiotic named Neoberninamycin**.** In another report by Kumari et al. [71], the crude yellow pigment of *Micrococcus* sp. OUS9 was active against both Gram (+) and Gram **(−**) bacteria, i.e*.*, *B. subtilis*, *K. pneumoniae. Salmonella* sp., *S. aureus*, *P. aeruginosa*, and *E. coli*, No literature has been available on *N. lactis* on its antibacterial activity except for the proposal for the creation of a new genus *Neomicrococcus* by Prakash et al. [70].

## Conclusion

Our attempt to isolate soil bacteria having antibacterial activity yielded encouraging results. There are 3 bacterial isolates, hitherto not reported for antibiotic activity have been characterized and identified as *B. aryabhattai*, *A. humicola*, and *N. lactis*. These isolates were tested against human pathogens, *S. aureus*, *E. coli*, *P. aeruginosa*, and *K. pneumoniae*. Among the pathogens tested, the Gram-negative bacteria *E. coli* was slightly resistant compared to other Gram-positive pathogens. Filter-sterilized culture filtrates had more antibacterial activity than autoclaved and proteinase K-treated culture filtrates. All isolates preferred different carbon and nitrogen sources for their enhanced activity. In our study, *B. aryabhattai* showed good antibacterial activity than the other two isolates. All the isolates showed broad-spectrum antibiotic activity, and further purification and standardization processes are required to compare the efficacy with all available antibiotics. We conclude that all these 3 bacteria are potential candidates for further research on their antibacterial properties.

## Data Availability

The datasets used and/or analyzed during the current study are available from the corresponding author on reasonable request.
